# Heading Frequency and Risk of Cognitive Impairment in Retired Male Professional Soccer Players

**DOI:** 10.1001/jamanetworkopen.2023.23822

**Published:** 2023-07-17

**Authors:** Shima Espahbodi, Eef Hogervorst, Tara-Mei Povall Macnab, Ahmed Thanoon, Gwen Sacha Fernandes, Bonnie Millar, Ashley Duncan, Maria Goodwin, Mark Batt, Colin W. Fuller, Gordon Fuller, Eamonn Ferguson, Tobias Bast, Michael Doherty, Weiya Zhang

**Affiliations:** 1 Academic Unit of Injury, Recovery and Inflammation Sciences, School of Medicine, University of Nottingham, Nottingham, UK; 2 Centre for Sport, Exercise and Osteoarthritis Research Versus Arthritis, University of Nottingham, Nottingham, UK; 3 Pain Centre Versus Arthritis, Academic Rheumatology, City Hospital, Nottingham, UK; 4 NCSEM, School of Sport, Exercise and Health Sciences, Loughborough University, Loughborough, UK; 5 Population Health Science, Bristol Medical School, University of Bristol, Bristol, UK; 6 NIHR Nottingham Biomedical Research Centre, University of Nottingham, Nottingham, UK; 7 National Institute for Health Research ARC EM, University of Nottingham, Nottingham, UK; 8 Colin Fuller Consultancy Ltd, Sutton Bonington, UK; 9 Centre for Urgent and Emergency Research, University of Sheffield, Sheffield, UK; 10 School of Psychology, University of Nottingham, Nottingham, UK; 11 National Institute for Health and Care Research Blood and Transplant Research Unit in Donor Health and Behaviour, University of Cambridge, Cambridge, UK; 12 Neuroscience@Nottingham, University of Nottingham, Nottingham, UK

## Abstract

**Importance:**

Although professional soccer players appear to be at higher risk of neurodegenerative disease, the reason remains unknown.

**Objective:**

To examine whether heading frequency is associated with risk of cognitive impairment in retired professional soccer players.

**Design, Setting, and Participants:**

A UK nationwide cross-sectional study was conducted between August 15, 2020, and December 31, 2021, in 459 retired male professional soccer players older than 45 years and registered with the Professional Footballers’ Association or a League Club Players’ Association.

**Exposure:**

Data on heading frequency in 3 bands—0 to 5, 6 to 15, and more than 15 times per match or training session and other soccer-specific risk factors, such as player position and concussion—were collected through a self-reported questionnaire.

**Main Outcomes and Measures:**

Cognitive impairment was defined using the Telephone Interview for Cognitive Status-modified as scores of less than or equal to 21. Hopkins Verbal Learning Test, verbal fluency, and independent activities of daily living were also assessed. Test Your Memory and physician-diagnosed dementia/Alzheimer disease were self-reported via the questionnaire. Adjusted odds ratios (AORs) with 95% CIs were calculated.

**Results:**

Of 468 retired male professional soccer players who completed questionnaires (mean [SD] age, 63.68 [10.48]; body mass index, 27.22 [2.89]), 459 reported heading frequency: 114 headed 0 to 5 times, 185 headed 6 to 15 times, 160 headed more than 15 times per match, and 125 headed 0 to 5 times, 174 headed 6 to 15 times, and 160 headed more than 15 times per training session during their careers. The prevalence of cognitive impairment was 9.78% (0-5 times), 14.78% (6-15 times), and 15.20% (>15 times) per match (*P* = .51). Compared with players reporting 0 to 5 headers per match, the AORs were 2.71 (95% CI, 0.89-8.25) for players reporting 6 to 15 headers per match and 3.53 (95% CI, 1.13-11.04) for players reporting more than 15 headers per match (*P* = .03 for trend). Corresponding AORs for heading frequency per training session were 2.38 (95% CI, 0.82-6.95) for those reporting 6 to 15, and 3.40 (95% CI, 1.13-10.23) for those reporting more than 15 in comparison with those who reported 0 to 5 (*P* = .03 for trend). Concussion involving memory loss was also associated with a greater risk of cognitive impairment (AOR, 3.16; 95% CI, 1.08-9.22). Similar results were observed with other cognitive tests and self-reported physician-diagnosed dementia/Alzheimer disease.

**Conclusions and Relevance:**

The findings of this study suggest that repetitive heading during a professional soccer career is associated with an increased risk of cognitive impairment in later life. Further study is needed to establish the upper threshold for heading frequency to mitigate this risk.

## Key Points

**Question:**

Is repetitive heading of the ball during a professional soccer career associated with an increased risk of cognitive impairment in male soccer players in their later lives?

**Findings:**

In this cross-sectional study, players with heading frequency more than 15 times per match or training session had more than a 3-fold risk of cognitive impairment compared with those with a heading frequency 0 to 5 times per match or training session. Similar results were observed with other cognitive tests noted with dementia and Alzheimer disease.

**Meaning:**

The findings of this study suggest that heading the ball in soccer is associated with a risk of cognitive impairment; the upper-frequency threshold for heading remains to be determined to mitigate this risk.

## Teaser

This cross-sectional study examines the prevalence of cognitive impairment in retired professional soccer players based on the frequency of heading within training sessions and matches.

## Introduction

In professional soccer players, repetitive brain macrotrauma and microtrauma and its long-term consequences on neurocognitive function, particularly when resulting from repetitive soccer heading,^1,2^ remain a concern. During normal play, unintentional overt head impacts between players involving head to head, body, and ground contact can result in traumatic brain injury (TBI) or concussion (mild TBI).^1^ While moderate to severe TBI is a risk factor for dementia across all ages, mild TBI increases dementia risk in individuals older than 65 years.^3^ Heading in soccer is an intentional part of the game but is now thought to cause insidious injury or subconcussive head trauma.^4,5,6^ Over a soccer player’s career, chronic subconcussive trauma from repetitive heading during matches and training may cause cumulative brain injury that increases the risk of neurodegenerative diseases including chronic traumatic encephalopathy and associated cognitive impairments.^7^

Two big data studies conducted in Scotland^8^ and France^9^ reported more than 3 times an increased risk of death with neurodegenerative diseases in former soccer players compared with age-matched general population controls. The same Scottish study also found that defenders had the highest risk (hazard ratio, 4.98; 95% CI, 3.18-7.79) and goalkeepers the lowest risk (hazard ratio, 1.83; 95% CI, 0.93-3.60) of neurodegenerative disease,^10^ implying that exposure to factors associated with nongoalkeeper positions increased the risk. Similar results were observed in the more recent Swedish study.^11^ However, data on players’ exposures to heading and concussion were not available from these historical big data studies. Therefore, further study in living soccer players into specific risk factors, such as soccer-specific repetitive heading, associated with neurologic consequences is still needed. We therefore undertook this study, aiming to examine, within retired professional soccer players, whether soccer-specific risk factors, primarily heading frequency, is associated with objectively measured cognitive function scores and self-reported physician diagnosis of dementia/Alzheimer disease. Our primary hypothesis was that repetitive heading would be associated with the risk of cognitive impairment and self-reported dementia in retired soccer players; all other factors were considered exploratory.

## Methods

### Study Design and Participants

This cross-sectional study was conducted between August 15, 2020, and December 31, 2021, in living, retired, professional male soccer players older than 45 years to examine their current cognitive status and retrospectively investigate their heading exposures and other soccer-specific risk factors during their early life careers. The study was approved by the East Midlands-Leicester Central Research Ethics Committee. Study results are reported following the Strengthening the Reporting of Observational Studies in Epidemiology (STROBE) reporting guideline. The detailed protocol of the Foot and Ankle Osteoarthritis and Cognitive Impairment in UK Soccer Players (FOCUS) study has been published.^12^ Questionnaires were posted to 878 retired professional male soccer players fulfilling the inclusion criteria of being between ages 40 and 100 years, registered with the Professional Footballers’ Association or a League Club Players’ Association who had participated in a previous University of Nottingham study and had consented to being contacted for future studies.^13^ Responders who completed the questionnaire and indicated willingness to undergo cognitive function tests provided consent and underwent the assessments via a structured telephone interview. No financial compensation was provided.

### Exposure

Our primary hypothesis was that heading frequency would be associated with a risk of cognitive impairment; all other soccer-specific factors were considered exploratory. Data on heading frequency, classified into 3 bands (0-5, 6-15, and >15 times per typical professional match and per typical training session), were collected via the FOCUS questionnaire.^12^ Heading exposure questions were designed in consultation with the FOCUS Patient and Public Involvement group of retired professional soccer players^12^ and were also in line with published data on heading in professional soccer.^14,15^ The FOCUS Patient and Public Involvement group advised that question responses be grouped into 3 categories according to heading frequencies (0-5, 6-15, and >15 times per typical match/training session) to ease recollection for participants and minimize the recall bias. The final questionnaire design was piloted with 15 retired players from the Professional Footballers’ Association and Notts County Football Club.^12^ Concussion from football was classified as yes/no; if yes, how many times and how many times the concussion involved (1) loss of consciousness, (2) loss of memory, and (3) hospitalization. A definition of concussion, derived from an established list of concussion signs and symptoms,^16^ was provided before questions on concussion. Other soccer-specific risk factors that were examined included playing positions, career duration (years), total number of matches played, and average training hours per week during their professional career.

### Outcomes

Cognitive function was the primary outcome, assessed using the Telephone Interview for Cognitive Status-modified (TICS-m), in which cognitive impairment is defined as a TICS-m score of 21 or lower.^17^ In addition, Hopkins Verbal Learning Test (HVLT), verbal fluency (VF), and independent activities of daily living (IADL) were assessed through the telephone interview.^12^ The cutoffs for these additional tests were established using a receiver operating characteristic curve analysis with physician diagnosis of dementia/Alzheimer disease as the reference. Receiver operating characteristic analyses demonstrated significant areas under the curve (AUC) for all cognitive tests against self-reported dementia (*P* < .001), with an optimal cutoff score of 20.5 (AUC, 0.90) for TICS-m, 14.5 (AUC, 0.98) for HVLT, 19.5 (AUC, 0.89) for VFT, 15.5 (AUC, 0.94) for IADL, and 35.5 (AUC, 0.92) for the Test Your Memory (TYM) test. Physician-diagnosed dementia/Alzheimer disease were self-reported through the questionnaire, which also included the TYM test.^18^

### Covariates

Covariate risk factors for dementia^19^ collected in the questionnaire included age, body mass index (BMI) (calculated as weight in kilograms divided by height in meters squared), smoking, alcohol consumption, hearing loss, and comorbidities. The Charlson Comorbidity Index score was calculated as a composite score of 17 diseases weighted by the severity of disease.^20^ In this study, the diseases included myocardial infarction/coronary heart disease, heart failure, peripheral vascular disease, stroke (major or transient), connective tissues disorders/rheumatic diseases, chronic obstructive pulmonary disease, peptic ulcer, liver diseases (mild, moderate, and severe), diabetes, kidney failure, HIV/AIDs, and cancer. Dementia/Alzheimer disease was excluded from this score as it was a secondary outcome of this study. Hearing loss was considered as it affects the test and is a risk factor for dementia.^19^ Information on educational level was collected during the telephone assessment using levels 0 to 8 and subsequently categorized as basic (none, primary, or secondary school), moderate (school certificate, O-level/general certificate of secondary education/standard grade, higher school certificate/A-level/highers/advanced highers, or technical/trade certificate), and high (diploma, degree, or postgraduate degree). Socioeconomic status (ie, index of multiple deprivation) in deciles was obtained according to post code.^21^

### Statistical Analysis

Continuous data are presented as mean (SD) or median (IQR) depending on whether they were normally distributed, whereas dichotomous and categorical data are presented as frequency and percentages. The analysis of variance (for mean) or Kruskal-Wallis (for median) test was used for continuous data depending on distribution, whereas the Pearson χ^2^ test was used for categorical or dichotomous data. The Spearman correlation coefficient test was used to examine the correlation between categorical variables. Prevalence of cognitive impairment was calculated from the number of players with a TICS-m score of 21 or lower divided by the total number of players in each exposure group. Odds ratios (ORs) and 95% CIs were calculated between groups using the lowest band of heading frequency as the reference for both matches and training sessions. Adjusted OR (AOR) was calculated with the adjustment for age, BMI, educational level (or socioeconomic status for self-reported dementia/Alzheimer disease and TYM), smoking, alcohol intake, hearing loss, and the Charlson Comorbidity Index score. Data on race were collected as part of the demographic data of the study to measure the selection bias (eg, responders vs nonresponders), but race was not adjusted for as it is not an established risk factor for dementia. Other soccer-specific risk factors, including playing position and concussion, were not adjusted for as they were related to heading frequency. The dose-response association was tested using *P* for trend, with heading frequency coded as 1 for 0 to 5, 2 for 6 to 15, and 3 for more than 15 in a single variable, and *P* for trend was calculated using the likelihood ratio test. A cross-table comparison was generated to determine the AORs for cumulative exposure when considering heading frequency per match and training together for each player. Stepwise linear regression analysis was also conducted to examine each test score (as a continuous outcome) and heading frequency, adjusted for the same group of confounding factors. All statistical analyses were performed using SPSS, version 28 (SPSS Inc). All tests were 2-sided, and significance was set at *P* < .05.

## Results

### Characteristics of Study Population

Of 878 posted questionnaires, 554 were returned, 468 were completed, and 459 reported heading frequency per match and training (Figure). Of those who responded to the questionnaire, 326 players completed the telephone assessment. Participants were all male, with a mean (SD) age of 63.68 (10.48) years and BMI of 27.22 (2.89). A comparison of nonresponders vs responders for both the questionnaire and telephone assessment samples demonstrated generally no significant difference for age, BMI, socioeconomic status, race, and comorbidities, confirming that both questionnaire and cognitive test samples were representative of the source population (eTable 1 in Supplement 1).

**Figure.**
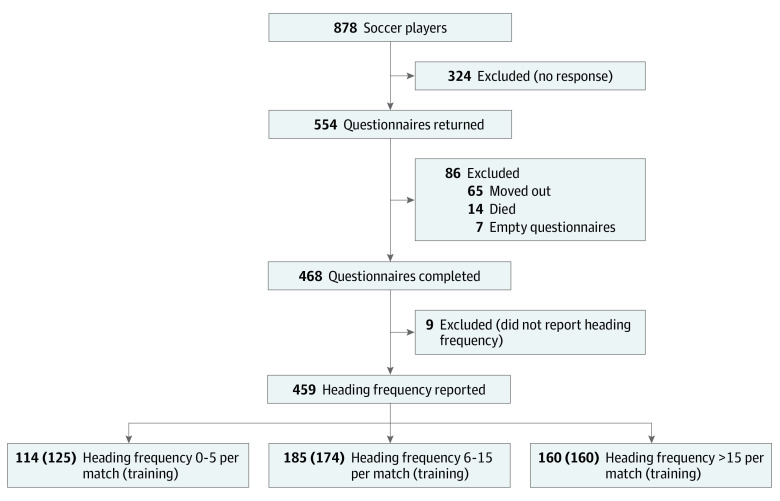
Study Population

As heading frequency per match was associated with heading frequency per training (Spearman correlation 0.69; *P* < .001), we used heading frequency per match as an index for heading exposure to compare study population characteristics across the different heading exposure groups (0-5, 6-15, and >15) (Table 1). Apart from age, heading frequency per training, training hours/week, and player position, no significant difference was seen among the 3 groups for other potential risk factors, such as BMI, socioeconomic status, educational level, and Charlson Comorbidity Index score. Apart from the TYM, the mean scores of other cognitive and function tests were not significantly different among the 3 heading bands. No self-reported dementia/Alzheimer disease was reported in the lowest heading band (0 of 114), but (5 of 185 [2.70%]) in the middle band and (8 of 160 [5.0%]) in the highest band were noted, with the difference being statistically significant (*P* = .048) (Table 1).

**Table 1.** Characteristics of the Study Population

Variable	Headings per match, No.			*P* value^a^
	0-5 (n = 114)	6-15 (n = 185)	>15 (n = 160)	
Age, mean (SD), y	66.57 (10.20)	63.72 (10.78)	61.57 (9.76)	<.001
BMI, mean (SD)	26.99 (3.17)	26.94 (3.10)	27.56 (3.43)	.18
Socioeconomic decile, mean (SD)	7.44 (2.58)	7.30 (2.42)	7.76 (2.28)	.26
Educational level 0-8, median (IQR)	3 (2-5)	3 (3-6)	4 (3-6)	.08
Charlson Comorbidity Index, median (IQR)	0 (0-1)	0 (0-1)	0 (0-1)	.23
Smoking, No. (%)^b^				
Never	91 (80.53)	152 (84.44)	126 (81.29)	.78
Past	18 (15.93)	23 (12.78)	26 (16.77)	
Current	4 (3.54)	5 (2.78)	3 (1.94)	
Alcohol use, No. (%)^b^				
Never	9 (9.68)	10 (6.17)	6 (4.44)	.53
Rarely	17 (18.28)	37 (22.84)	27 (20.00)	
Sometimes	49 (52.69)	90 (58.02)	80 (59.26)	
Almost every day	18 (19.35)	21 (12.96)	22 (16.30)	
Hearing loss, No. (%)	10 (8.77)	20 (10.81)	17 (10.63)	.84
Heading frequency per training, No. (%)^b^				
0-5	87 (76.99)	34 (18.38)	3 (1.89)	<.001^c^
6-15	21 (18.58)	111 (60.0)	41 (25.79)	
>15	5 (4.42)	40 (21.62)	115 (73.33)	
Career years, mean (SD)	14.50 (5.75)	13.47 (5.21)	13.63 (5.42)	.26
Total matches played, mean (SD)	492 (260)	449 (223)	479 (250)	.28
Training hours/week, mean (SD)	13.85 (4.63)	13.98 (4.66)	15.19 (5.92)	.049
Position played, No. (%)				
Goalkeeper	37 (32.46)	0	0	<.001^c^
Midfield	27 (23.68)	44 (23.78)	16 (10.19)	
Forward	35 (30.70)	60 (32.43)	47 (29.94)	
Defender	15 (13.16)	81 (43.78)	94 (59.87)	
Concussion from football, No. (%)	41 (36.28)	68 (37.16)	75 (47.77)	.08
Self-reported outcomes, No. (%)				
Dementia/Alzheimer disease	0	5 (2.70)	8 (5.0)	.048
TYM, mean (SD)	38.06 (3.09)	37.45 (3.32)	36.78 (5.23)	.045
Cognitive and function tests, mean (SD)				
TICS-m	26.54 (4.03)	26.43 (5.14)	25.70 (4.73)	.35
HVLT	22.36 (5.29)	22.53 (6.52)	22.56 (5.82	.97
VF	21.19 (5.27)	21.10 (6.25)	21.89 (5.70)	.53
IADL	15.43 (1.72)	15.33 (1.67)	14.95 (2.70)	.23

Abbreviations: BMI, body mass index (calculated as weight in kilograms divided by height in meters squared); HVLT, Hopkins Verbal Learning Test; IADL, instrumental activities of daily life; TICS-m, Telephone Interview for Cognitive Status-modified; TYM, Test Your Memory; VF, verbal fluency.

^a^ *P* values represent overall difference/trend among the 3 heading frequency groups. They were calculated from the analysis of variance test for mean, Kruskal-Wallis test for median, and Pearson χ^2^ test for categorical or dichotomous variables, unless otherwise specified.

^b^ Percentages are based on the number of people who answered the question, so the total number in each category is not necessarily equal to the total number of people in that category.

^c^ Spearman correlation test.

### Heading Frequency and Risk of Cognitive Impairment

The prevalence of cognitive impairment was 9.78% (0-5 times), 14.78% (6-15 times), and 15.20% (>15 times) per match (*P* = .51). Compared with the lowest heading frequency per match (0-5), the AOR was 2.71 (95% CI, 0.89-8.25) for players reporting 6 to 15 headings per match and 3.53 (95% CI, 1.13-11.04) for players reporting more than 15 headings per match. A dose-response association was observed (*P* = .03 for trend) (Table 2). Similarly, compared with 125 players reporting the lowest heading frequency per training (0-5), the AOR was 2.38 (95% CI, 0.82-6.95) for 174 players reporting 6 to 15 headings per training and 3.40 (95% CI, 1.13-10.23) for 160 players reporting more than 15 headings per training session; a dose-response association was observed (*P* = .03 for trend) (Table 2).

**Table 2.** Heading Frequency and Cognitive Impairment^a^

Headings, No.	Cognitive impairment, No.	Total headings, No.	OR (95% CI)	AOR (95% CI)^b^
**Per match**				
0-5	8	114	1 [Reference]	1 [Reference]
6-15	17	185	1.58 (0.65-3.87)	2.71 (0.89-8.25)
>15	19	160	1.64 (0.68-3.94)	3.53 (1.13-11.04)
*P* value for trend			.31	.03
**Per training**				
0-5	7	125	1 [Reference]	1 [Reference]
6-15	19	174	2.04 (0.82-5.08)	2.38 (0.82-6.95)
>15	18	160	2.11 (0.84-5.31)	3.40 (1.13-10.23)
*P* value for trend			.14	.03

Abbreviations: AOR, adjusted odds ratio; OR, odds ratio.

^a^ Cognitive impairment was defined as a Telephone Interview for Cognitive Status-modified score less than or equal to 21.

^b^ Adjusted for age, body mass index, educational level, smoking, alcohol intake, hearing loss and Charlson Comorbidity Index score.

When considering heading frequency per match and training together for each player, the risk of cognitive impairment increased with the cumulative heading frequency; for example, compared with players who headed 0 to 5 times on both occasions, the AOR was 4.29 (95% CI, 1.14-16.10) for players who headed 6 to 15 times and 4.71 (95% CI, 1.20-18.45) for players who headed more than 15 times on both occasions (Table 3).

**Table 3.** Cognitive Impairment by Heading Frequency per Match and Training^a^

No. of headings per training	No. of headings per match, AOR (95% CI)^b^		
	0-5	6-15	>15
0-5	1 [Reference]	2.03 (0.38-10.91)	NA
6-15	2.03 (0.38-10.91)	4.29 (1.14-16.10)	3.45 (0.75-15.85)
>15	NA	3.45 (0.75-15.85)	4.71 (1.20-18.45)

Abbreviations: AOR, adjusted odds ratio; NA, not applicable.

^a^ Cognitive impairment was defined as a Telephone Interview for Cognitive Status-modified score less than or equal to 21.

^b^ Adjusted for age, body mass index, educational level, smoking, alcohol intake, hearing loss, and Charlson Comorbidity Index score.

### Other Risk Factors

The odds of having cognitive impairment tended to increase according to playing position, being lowest for goalkeepers (AOR 1, reference), followed by midfielders (AOR, 1.48; 95% CI, 0.22-9.76), forwards (AOR, 1.92; 95% CI, 0.32-11.45), and defenders (AOR, 3.16; 95% CI, 0.54-18.62) after the adjustment, although the results were not statistically significant (*P* = .09 for trend) (eTable 2 in Supplement 1). While the odds were not increased with career length (years), total matches played, or average training hours/week, the odds were increased with soccer-specific concussion involving loss of memory (AOR, 3.16; 95% CI, 1.08-9.22) (eTable 2 in Supplement 1).

### Other Outcomes

Table 4 reports the results for other cognitive impairment outcomes. After adjusting for age, BMI, educational level (or socioeconomic status for self-reported outcomes), smoking, alcohol consumption, hearing loss, and the Charlson Comorbidity Index score, increased heading per match and heading per training were both associated with cognitive impairment defined with a TICS-m score of 23 or lower (another recommended cutoff),^22^ IADL, TYM, and self-reported dementia/Alzheimer disease. The results were supported by the linear regression analysis with TICS-m, IADL, and TYM, but not HVLT and VF associated with heading frequency per match and training after adjusting for the same group of confounding factors (eTable 3 in Supplement 1).

**Table 4.** Heading Frequency and Other Cognitive Impairment Outcomes

Headings, No.	Other outcome, AOR (95% CI)^a^					
	TICS-m ≤23^b^	HVLT ≤14.5	VF ≤19.5	IADL ≤15.5	TYM ≤35.5	Dementia/Alzheimer disease
**Per match**						
0-5	1 [Reference]	1 [Reference]	1 [Reference]	1 [Reference]	1 [Reference]	NA^c^
6-15	2.32 (1.00-5.38)	3.06 (0.88-10.62)	1.26 (0.60-2.61)	2.24 (0.90-5.56)	2.21 (1.01-4.85)	1 [Reference]
>15	3.19 (1.31-7.30)	2.68 (0.74-9.76)	1.18 (0.56-2.50)	3.54 (1.42-8.83)	2.88 (1.27-6.56)	6.91 (0.92-52.01)
*P* value for trend	.01	.16	.70	.007	.01	.007
**Per training session**						
0-5	1 [Reference]	1 [Reference]	1 [Reference]	1 [Reference]	1 [Reference]	1 [Reference]
6-15	3.32 (1.44-7.65)	2.23 (0.69-7.20)	1.16 (0.57-2.36)	2.26 (0.93-5.48)	1.62 (0.78-3.38)	2.89 (0.25-34.05)
>15	3.03 (1.28-7.21)	2.20 (0.65-7.43)	1.24 (0.60-2.56)	3.88 (1.56-9.64)	2.38 (1.14-5.00)	17.74 (1.39-226.75)
*P* value for trend	.02	.22	.58	.003	.02	.02

Abbreviations: AOR, adjusted odds ratio; HVLT, Hopkins Verbal Learning Test; IADL, instrumental activities of daily life; NA, not applicable; TICS-m, Telephone Interview for Cognitive Status-modified; TYM, Test Your Memory; VF, verbal fluency.

^a^ Adjusted for age, body mass index, educational level, smoking, alcohol intake, hearing loss, and Charlson Comorbidity Index score.

^b^ A cutoff level of 23 or less was included here as a sensitivity analysis. The results were similar to those based on the cutoff level of 21 or less.

^c^ Although there were no dementia/Alzheimer disease cases at level 0 to 5, the overall analysis demonstrated a trend with an average AOR of 5.89 (95% CI, 1.55-22.42) and *P* = .007 for trend per grade increase.

## Discussion

To our knowledge, this is the first study primarily focusing on heading frequency during soccer players’ professional career period and its long-term consequences, specifically cognitive impairment and self-reported physician-diagnosed dementia/Alzheimer disease in living retired professional soccer players. The main findings are (1) both match and training heading frequency during a soccer player’s professional career were associated with a risk of cognitive impairment in the player’s later life, (2) the associations were dose dependent, and (3) the findings were consistent across TICS-m, IADL, TYM, and self-reported physician-diagnosed dementia/Alzheimer disease.

Our results are generally consistent with those of the previous Football’s Influence on Lifelong Health and Dementia Risk (FIELD) study,^10^ which did not include any direct measure on heading, but instead used playing position as a surrogate for head impact exposure. Neurodegenerative disease risk was highest for defenders (5-fold) and lowest for goalkeepers (1.83-fold) compared with population controls. The authors concluded that exposure to factors associated with outfield (ie, nongoalkeeper) playing positions contributed to the increased risk, implying that head impacts/headings were the missing accountable factor^9^ since TBI risk and heading exposure varied by field-playing position.^23^ This is supported by our study noting that defenders headed the most, followed by forwards, midfielders, and goalkeepers. Our study recorded self-reported heading frequency and documented that heading during matches and training is a significant risk factor for cognitive impairment and self-reported physician-diagnosed dementia/Alzheimer disease. We also found the risk of cognitive impairment tended to increase in the sequence from goalkeeper to midfielder, forward, and defender positions, although the trend was not statistically significant (*P* = .09 for trend). This may occur because playing position is a group risk measure of head impact and, hence, less sensitive to change. Moreover, unlike the FIELD study, which used the general population as the control, we used goalkeeper position as the control to calculate the OR, which may have underestimated the risk. This approach was used simply because it was impossible to collect heading frequency data for the general population controls.

Evidence suggests that repetitive subconcussive trauma from heading the ball has a cumulative damaging effect on brain health^24,25,26,27,28^ and this is supported by chronic traumatic encephalopathy noted on autopsies of athletes who participated in contact sports, including former soccer players.^29,30^ In active players, heading the ball just 20 times during practice sessions can cause immediate and measurable effects on cognitive ability and function.^31^ Therefore, it would seem advisable to reduce exposure to head impacts and repetitive subconcussive head injuries. Our study used 3 broad bands for heading frequency: 0 to 5, 6 to 15, and more than 15 per match and training session. These values were adopted after consultation with the Patient and Public Involvement group and published data^13,14^ relating to heading exposure in professional soccer. The results suggested that heading 6 to 15 times per match or training session may not be significantly different from heading 0 to 5 times per match or training session, whereas heading more than 15 times per match or training session would lead to a significant increase in risk over heading 0 to 5 times on either occasion. Moreover, the risk is accumulative between match and training. Heading 6 to 15 times alone on only one occasion may not make any significant difference, whereas heading 6 to 15 times on both occasions would double the risk of cognitive impairment. Similarly, heading more than 15 times on only one occasion but not the other may not make any significant difference, whereas heading more than 15 times in both match and training sessions would make the risk 5 times greater than heading 0 to 5 times on both occasions (Table 3).

Soccer players are exposed to chronic TBI from 2 main routes: concussions from head collisions (head to head, head to body, and head to goalpost)^1^ and repetitive subconcussive impacts from heading the ball, which is a unique aspect of soccer.^32^ Until recently, most studies investigating neurodegenerative and neurocognitive disease outcomes in soccer have focused on concussion, which may have the propensity to alter brain structure and blood flow.^33^ Previous reviews suggested that soccer-specific concussions are similar to those reported in the wider sports liteature,^34^ with weak or no evidence of associations between concussions from soccer and long-term neurologic or cognitive deficits.^16,35,36^ However, most of these studies recruited young active players who would be too young to show late cognitive effects and, therefore, are not suitable for comparison with our current findings. Players in our study were born between 1936 and 1976, with a mean age of 63.68 years and starting to have cognitive impairment; hence, they were more suitable for assessing potential long-term cognitive deficits. Concussion alone (yes/no) was not associated with cognitive impairment, whereas concussion involving loss of memory was associated with cognitive impairment. This may be due to diagnostic uncertainty rather than the severity of the concussion, especially when participants were asked to self-diagnose this condition according to a given list of established concussion symptoms.^15^

### Limitations

There are several limitations of this study. First, it was a cross-sectional and retrospective design. However, such a design allowed us to examine the players in their later life for cognitive impairment and to retrospectively investigate heading exposures during their early life when they were professional soccer players. As it is unlikely they would have had any cognitive impairment or dementia during their career period, the association identified from this study is likely to be temporal. Second, retrospective self-reported questionnaires may be subject to recall bias,^37,38^ as some players may find it challenging to estimate the precise number of headings per match or training session in the past. To minimize this bias, we used 3 broad categories for heading frequency: 0 to 5, 6 to 15, and more than 15 times, which enabled players to provide a rank ordering for heading frequency^14^ rather than a precise number, which is shown to have sufficient accuracy. Third, of the 326 players who completed cognitive testing, only 44 had cognitive impairment as defined by the TICS-m cutoff score of 21 or lower. Although the test sample was representative of the 878 source population and not substantially different from the questionnaire sample (eTable 1 in Supplement 1), this low prevalence may challenge the adjustment for multiple confounding factors. However, we used the Charlson Comorbidity Index as a composite for several comorbidities to keep the confounding factors to a minimum in the model and stepwise selection for risk factors to optimize the model performance as appropriate. The consistent results across different exposures (eg, heading frequency between match and training sessions), outcomes (eg, TICS-m, IADL, TYM, and self-reported dementia/Alzheimer disease), and regression models (logistic and linear) also support the findings. Fourth, due to the COVID-19 pandemic, we had to use telephone assessments and not the originally planned in-person neurologic and cognitive assessments.^12^ However, the TICS-m is a validated and well-established telephone test for cognitive function in older adults. In addition to ensuring there were no hearing problems before each telephone assessment (“Do you normally wear a hearing aid? Please make sure it is switched on”), we also adjusted for hearing loss as reported, which is a major confounding factor that may affect the telephone assessment.^19^ Due to only 13 self-reported cases of physician-diagnosed dementia, the results and conclusion pertaining to these cases should be interpreted with caution.

## Conclusions

The findings of this cross-sectional study suggest that repetitively heading the ball is a risk factor for cognitive impairment and self-reported dementias in retired UK soccer players. Further study is required to identify a safe maximum heading frequency per match and training session to mitigate this risk.

## Notes

Supplement 1. **eTable 1.** Comparison Between Responders Versus Nonresponders of the Study **eTable 2.** Other Soccer-Specific Risk Factors and Cognitive Impairment (TICS-m ≤21) **eTable 3.** Heading Frequency and Cognitive Test Scores—Linear Regression Model

Supplement 2.
Data Sharing Statement
