# Errors in the Byline

**DOI:** 10.1001/jamanetworkopen.2023.30818

**Published:** 2023-08-11

In the Original Investigation titled “Heading Frequency and Risk of Cognitive Impairment in Retired Male Professional Soccer Players,”^1^ published July 17, 2023, there was an error in the byline. The listings given as “Tobias Bast, MD” and “Michael Doherty, PhD” should have been “Tobias Bast, PhD” and “Michael Doherty, MD.” This article has been corrected.^1^

## References

[1] Espahbodi S, Hogervorst E, Macnab TMP, . Heading frequency and risk of cognitive impairment in retired male professional soccer players. JAMA Netw Open. 2023;6(7):e2323822. doi:10.1001/jamanetworkopen.2023.23822 37459095 PMC10352859

