# Supplementary material for: Heading Frequency and Risk of Cognitive Impairment in Retired Male Professional Soccer Players
Source: JAMA Netw Open. 2023 Jul 17;6(7):e2323822. doi: 10.1001/jamanetworkopen.2023.23822 (PMC10352859; doi:10.1001/jamanetworkopen.2023.23822)
Supplement: Supplement 1. — eTable 1. Comparison Between Responders Versus Nonresponders of the Study eTable 2. Other Soccer-Specific Risk Factors and Cognitive Impairment (TICS-m ≤21) eTable 3. Heading Frequency and Cognitive Test Scores—Linear Regression Model [file jamanetwopen-e2323822-s001.pdf]

Supplementary Online Content

Espahbodi S, Hogervorst E, Macnab TMP, et al. Heading frequency and risk of cognitive impairment in retired male professional soccer players. *JAMA Netw Open*. 2023;6(7):e2323822. doi:10.1001/jamanetworkopen.2023.23822

eTable 1. Comparison Between Responders Versus Nonresponders of the Study

eTable 2. Other Soccer-Specific Risk Factors and Cognitive Impairment (TICS-m ≤21)

eTable 3. Heading Frequency and Cognitive Test Scores—Linear Regression Model

This supplementary material has been provided by the authors to give readers additional information about their work.

© 2023 Espahbodi S et al. *JAMA Network Open.*

eTable 1. Comparison between responders versus nonresponders of the study

	Questionnaire			Cognitive test		
	Responders	Non-responders	P value*	Responders	Non-responders	P value*
No. participants	**468**	**410**		**326**	**552**	
Age (years), mean (SD)	63.68 (10.48)	63.09 (12.27)	.441[a]	63.29 (10.00)	63.47 (12.08)	.815[a]
BMI, mean (SD)	27.22 (2.89)	27.48 (3.13)	.215 [a]	27.12 (2.89)	27.47 (3.07)	.096[a]
SES, mean (SD)	7.50 (2.41)	7.27 (2.45)	.232 [a]	7.62 (2.36)	7.25 (2.47)	.047[a]
Race, n (%)						
White	376 (80.34)	310 (92.81)	.080[b]	263 (93.93)	423 (93.58)	.018[b]
Black	13 (2.78)	7 (2.10)		12 (4.29)	8 (1.77)	
Other	9 (1.92)	17 (5.09)		5 (1.79)	21 (4.65)	
Comorbidities, n (%)						
CV	100 (21.37)	121 (29.51)	.699[b]	63 (19.33)	158 (28.62)	.366[b]
MSK	18 (3.85)	15 (3.66)		12 (3.68)	21 (3.80)	
Cancer	36 (7.69)	21 (5.12)		25 (7.67)	32 (5.80)	

CV: Cardio/cerebro vascular diseases including hyper cholesterol, hypertension, heart attack/angina, stroke and diabetes.
MSK: musculoskeletal diseases including osteoarthritis, fibromyalgia, irritable bowel syndrome and chronic widespread pain.
SES: Index of multiple deprivation was measured according to postcode.
BMI: Body mass index (kg/m2)
a = T -Test
b = Chi-Square test

© 2023 Espahbodi S et al. *JAMA Network Open.*

eTable 2. Other soccer-specific risk factors and cognitive impairment (TICS-m≤21)

	OR (95%CI)	AOR (95%CI)
Position played		
Goalkeeper	1 (reference)	1 (reference)
Midfield	1.34 (0.26, 6.94)	1.48 (0.22, 9.76)
Forward	2.03 (0.43, 9.52)	1.92 (0.32, 11.45)
Defender	2.07 (0.45, 9.49)	3.16 (0.54, 18.62)
P value for trend	0.232	0.089
Career years	1.00 (0.95, 1.07)	0.97 (0.89, 1.04)
Total matches played	1.000 (0.999, 1.001)	1.000 (0.998, 1.002)
Training hours per week	1.01 (0.95, 1.08)	1.04 (0.96, 1.12)
Concussion from football		
Yes/No	0.79 (0.41, 1.52)	1.03 (0.46, 2.31)
No. concussions	0.93 (0.68, 1.28)	0.99 (0.71, 1.38)
With memory loss	1.46 (0.61, 3.52)	3.16 (1.08, 9.22)

TICS-m: Telephone Interview for Cognitive status modified; OR: odds ratio; CI: confidence interval; AOR: odds ratio adjusted for age, body mass index, education, smoking, alcohol intake, hearing loss and Charlson comorbidity score.

© 2023 Espahbodi S et al. *JAMA Network Open.*

eTable 3. Heading frequency and cognitive test scores – linear regression model

	β (95%CI)				
	TICS-m	HVLT	VF	IADL	TYM
Heading frequency/match	-1.09 (-1.77, -0.41)	-0.66 (-1.47, 0.16)	0.02 (-0.84, 0.86)	-0.50 (-0.83, -0.16)	-1.05 (-1.75, -0.35)
p	0.002	0.114	0.956	0.004	0.003
Heading frequency/training	-0.95 (-1.61, -0.29)	-0.39 (-1.19, 0.41)	-0.03 (-0.85, 0.79)	-0.54 (-0.86, -0.21)	-1.11 (-1.77, -0.41)
p	0.005	0.335	0.940	0.001	0.002

TICS-m: Telephone Interview for Cognitive status modified; HVLT: Hopkins Verbal Learning Test; VF: verbal fluency; IADL: Instrumental Activities of Daily Life; TYM: Test Your Memory; β: regression coefficient, adjusted for age, BMI, education, smoking, alcohol intake, hearing loss and Charlson comorbidity score through the stepwise linear regression model; CI: confidence interval.

© 2023 Espahbodi S et al. *JAMA Network Open.*
