# Supplementary material for: Heading Frequency and Risk of Cognitive Impairment in Retired Male Professional Soccer Players
Source: JAMA Netw Open. 2023 Jul 17;6(7):e2323822. doi: 10.1001/jamanetworkopen.2023.23822 (PMC10352859; doi:10.1001/jamanetworkopen.2023.23822)
Supplement: Supplement 2. — Data Sharing Statement [file jamanetwopen-e2323822-s002.pdf]

Data Sharing Statement

Espahbodi. Heading Frequency and Risk of Cognitive Impairment in Retired Male Professional Soccer Players. *JAMA Netw Open*. Published July 17, 2023.
doi:10.1001/jamanetworkopen.2023.23822

Data
Data available: Yes
Data types: Deidentified participant data
How to access data: Data are available upon request to Professor Weiya Zhang as weiya.zhang@nottingham.ac.uk
When available: With publication

Supporting Documents
Document types: None

Additional Information
Who can access the data: Researchers whose proposed use of the data has been approved
Types of analyses: For research purpose only
Mechanisms of data availability: With a signed data access agreement
